# Overlooked sources of inspiration in biomimetic research

**DOI:** 10.1038/s41598-025-11703-6

**Published:** 2025-07-15

**Authors:** Jindong Zhang, Simon Baeckens, Raoul Van Damme, Kristina Wanieck

**Affiliations:** 1 Working Group Biomimetics, Faculty of Applied Informatics, Technology Campus Freyung, Deggendorf Institute of Technology, Freyung, Germany; 2 Department of Biology, Laboratory of Functional Morphology, University of Antwerp, Antwerp, Belgium; 3 Department of Biology, Ghent University, Ghent, Belgium

**Keywords:** Bioinspiration, Biomimicry, Biodiversity, Taxonomy, Evolutionary biology, AI, Biomimetics, Evolutionary theory, Taxonomy

## Abstract

Biomimetics draws inspiration from biological organisms, yet only a small fraction of Earth’s biodiversity has been explored for innovation. This study examines the biological models used in biomimetic research. Using GPT-4o, we analyzed 74,359 publications and identified 31,776 biological models, revealing distinct taxonomic distribution patterns. Our findings highlight a reliance on a narrow set of animal taxa; fewer than 23% of identified models were resolved at the species level—corresponding to 1,604 species; and broad taxonomic classifications (e.g., phylum, class) were more frequently cited. Despite the rapid growth of biomimetics, the exploration of new model taxa falters. Researchers tend to focus on one model per study, potentially limiting the field’s capacity to leverage evolutionary insights. To promote diversity and innovation in biomimetics, we advocate for stronger collaboration with biologists to integrate underutilized yet well-researched taxa, specify biological inspirations at the species level to enhance evolutionary insights, and—where appropriate—incorporate multiple models, enabling the use of comparative methods.

**Supplementary Information:**

The online version contains supplementary material available at 10.1038/s41598-025-11703-6.

## Introduction

Biomimetics drives innovation by translating biological principles into engineering and design solutions. The term “biomimetics” itself underscores that biology forms the foundation of this imitation process; understanding biological models is thus essential. With approximately 1.2 million documented species and an estimated 9 million eukaryotic species in total^1^, Earth’s biodiversity represents a nearly inexhaustible reservoir of potential inspiration. Well-known examples such as Velcro inspired by burdock burrs^2^, wind turbines modeled after humpback whale fins^3,4^, and adhesives inspired by gecko’s feet^5–7^ illustrate how biological strategies can be leveraged. The biomimetics research field is often subdivided into different branches, such as structural biomimetics (replicating the multiscale architectures and fabrication strategies of biological materials)^8^, functional biomimetics (emulating underlying mechanisms, processes, or behaviors)^9^, and extreme biomimetics (drawing inspiration from organisms thriving under exceptional environmental constraints)^10^. As the field advances, increasing numbers of chemists, engineers, and materials scientists recognize the value of seeking inspiration from biological systems to address their challenges^11^. However, are researchers truly making full use of the vast and varied library of biological diversity? Or have they been fixating on a limited number of eye-catching species? Have they embraced a fundamental principle of biology: that the study of *variation* is imperative to our understanding of (organismal) design^12^?

Some groups of organisms attract our attention more than others. This “taxonomic bias” is widespread in literary works^13,14^ and cinematography^15^, but also in scientific research, such as conservation biology^16,17^ and animal behavior studies^18^. The extent of taxonomic bias in biomimetic research has not yet been fully examined, but several authors have expressed their worry about the field’s fixation on a few iconic species or groups^19–21^. Analyses on small subsets of biomimetics publications support the image of a myopic choice of inspirational species^22–24^. If researchers draw inspiration from only a limited range of taxa, many valuable biological strategies may remain overlooked, ultimately constraining the field’s innovative potential. At the same time, biology does not unfold in a vacuum. Every species—inevitably including those applied in biomimetics—arose under distinct genetic and environmental pressures, resulting in substantial variation both within and across taxa^25,26^. This raises the question of whether biomimetics fully capitalizes on these evolutionary nuances. For instance, focusing on a single gecko lineage may overlook other lineages with subtly different adhesive mechanisms shaped by their habitats^27^. It has been argued that a broader, comparative approach could reveal design strategies molded by distinct selective pressures, potentially expanding the scope and efficacy of biomimetic solutions^28^. However, whether biomimetics has come to embrace this recommendation is uncertain.

Here, we assess the taxonomic breadth and the use of comparative analyses in biomimetics by examining the titles, keywords, and abstracts of 74,359 relevant publications. Previous studies have not comprehensively analyzed the field, partly due to its sheer scale—manually reviewing tens of thousands of biomimetic studies for details on model species is technically challenging. However, the rapid advancements in artificial intelligence (AI), particularly large language models (LLMs), offer new avenues to address this challenge. For instance, Carniel et al. applied ChatGPT as an information-extracting tool for topic clustering in biomimetics research^29^, while Yeter and Le Ferrand explored ChatGPT’s capacity to assist users in applying biomimicry principles to technological challenges^30^. Building on these developments, this study employs GPT-4o (by OpenAI), a widely used commercial LLM, in combination with fine-tuned prompts and stepwise processing. Our objectives are to (i) systematically analyze all relevant biomimetic-related publications on an individual basis; (ii) determine whether each publication explicitly declares the biological source of inspiration—allowing even single occurrences of model organisms to be detected; (iii) conduct a comprehensive taxonomic analysis of all biological models used in biomimetics; and (iv) examine whether publications have used insights from evolutionary biology on how the environment shapes biological design—by considering their use of comparative or multi-model approaches. Through this study, we aim to uncover underlying taxonomic patterns and potential biases in biomimetics using precise, quantitative data, thereby addressing a long-standing gap in the field.

## Results

### The biodiversity of biomimetics

**Fig. 1:**
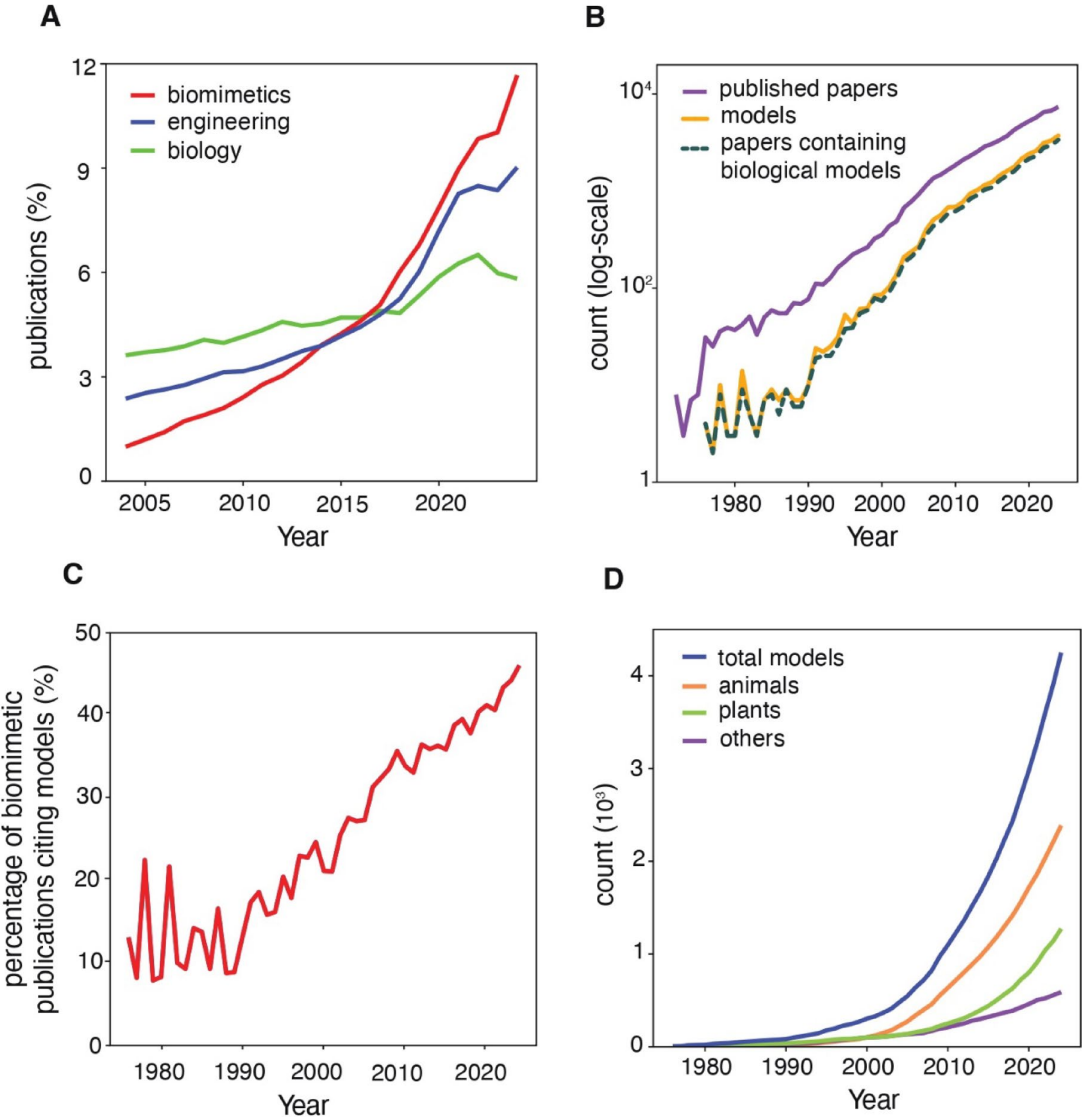
Temporal trends of biological models in biomimetics. (**A**) Percentage of publications in biomimetics, engineering, and biology from 2004 to 2024, calculated based on total publications within each field. Data retrieved from Web of Science on February 22, 2025. “Biology (WoS Category)” and “Engineering (WoS Category)” were queried directly, while biomimetics was queried using “biomim* (Topic) OR bioinspir* (Topic)”. (**B**) Annual publication trends of 74,359 retrieved papers, with the number of model counts per year and the number of papers citing biological models. (**C**) Annual percentage of biomimetic publications that explicitly cite biological models, illustrating the proportion of studies that declare a biological source of inspiration. (**D**) Cumulative count of newly introduced distinct models by taxonomic group. Models are counted only in their first year of citation. “others” comprises Bacteria, Fungi, Protista, Archaea, and Viruses.

By analyzing the publication trends, we found that the field of biomimetics has been growing at a staggering rate over the past 20 years, its growth trajectory closely mirrors that of the engineering field but is even faster—particularly with a surge in publication volume over the last two years (Fig. 1A). Using the Web of Science (WoS) query (“biomim*” (Topic) OR “bioinspir*” (Topic)), we retrieved 74,359 publications spanning 1972 to 2025. Of these, 5,038 (6.8%) were review papers that contained references to biological models, which were excluded from further analysis. In 28,333 publications (38.1%), at least one valid biological model was identified, yielding a total of 31,776 biological models—these model-containing publications form the foundation of our analysis. The excluded papers primarily comprise theoretical studies, framework proposals, or tool development work in biomimetics, which did not include identifiable biological models. When comparing biomimetics publication volume and use of biological models, both remained low until 1990 but increased sharply thereafter (Fig. 1B). Over time, the proportion of papers citing biological models increased from about 13% during biomimetics’ first decade (1976–1985: 52 papers with models among 403 papers) to nearly 41% in the most recent decade (2015–2024: 20,921 papers with models across 50,723 papers), highlighting a growing focus on biological model utilization (Fig. 1C).

**Fig. 2:**
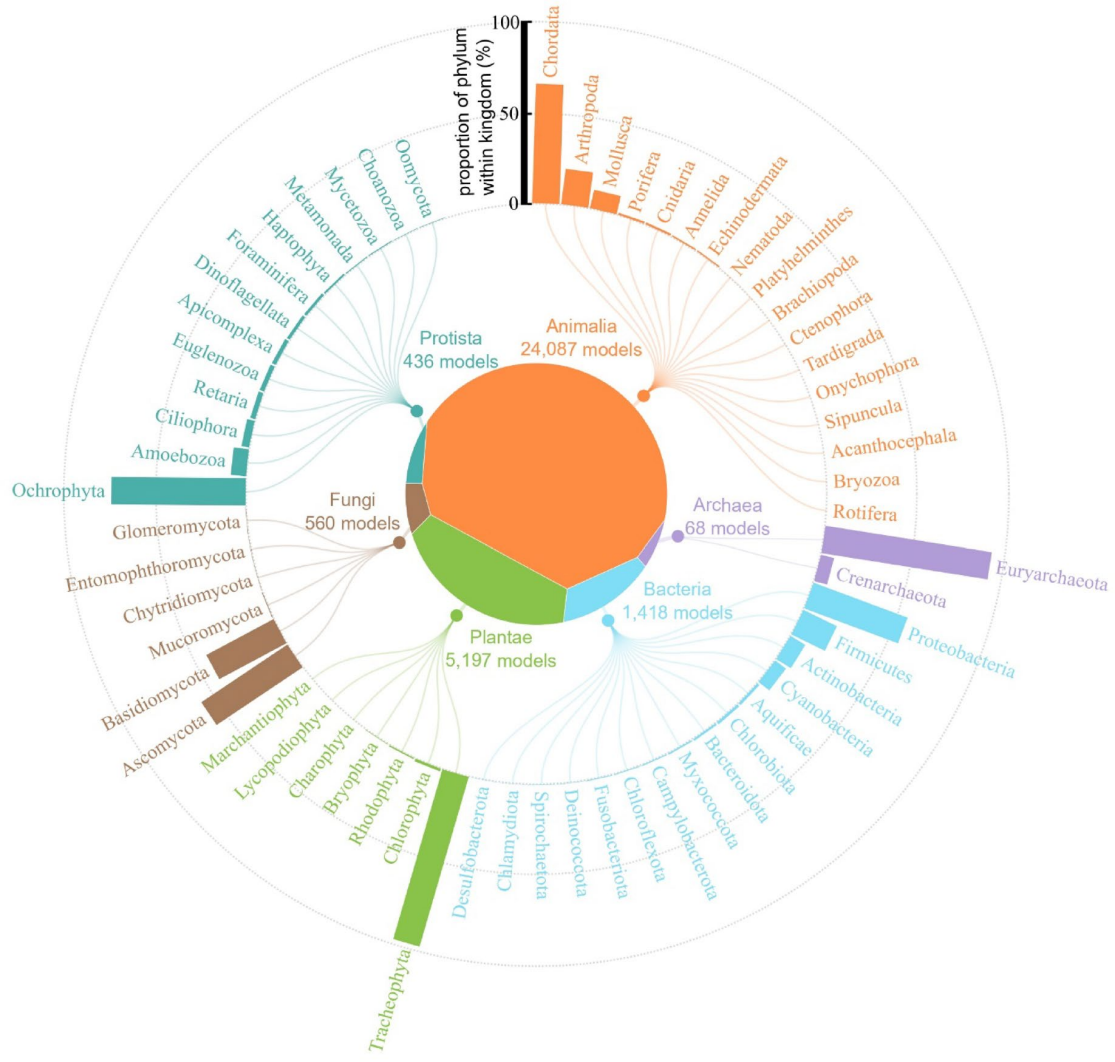
Phylum-level distribution of identified models. The center treemap illustrates the proportion of total models attributed to each of the six kingdoms (viruses excluded), while the circular barplot shows the percentage of each phylum within its respective kingdom. Only phyla with at least one identified model are labeled.

Analysis of the 31,776 identified biological models reveals that biomimetic research draws inspiration from all six kingdoms, as well as from viruses. While plants (kingdom Plantae) were relatively popular in early biomimetic research, animals (kingdom Animalia) became the dominant source of inspiration near the turn of the century (Fig. 1D). Their prominence has further increased over the last decade—by the end of 2024, animal-based models account for over 75% of all biological models cited in biomimetic research, while plants make up approximately 16% (Fig. 2). The other groups (Bacteria, Fungi, Protista, Archaea, and Viruses) have always played a less influential role. At the phylum level, most attention is given to chordates (Chordata—vertebrate animals), arthropods (Arthropoda—invertebrates such as insects, scorpions, spiders, crustaceans), molluscs (Mollusca—invertebrates such as snails, bivalves, octopuses) and vascular plants (Tracheophyta—“higher” plants such as ferns, conifers, and flowering plants) (Fig. 2).

**Fig. 3:**
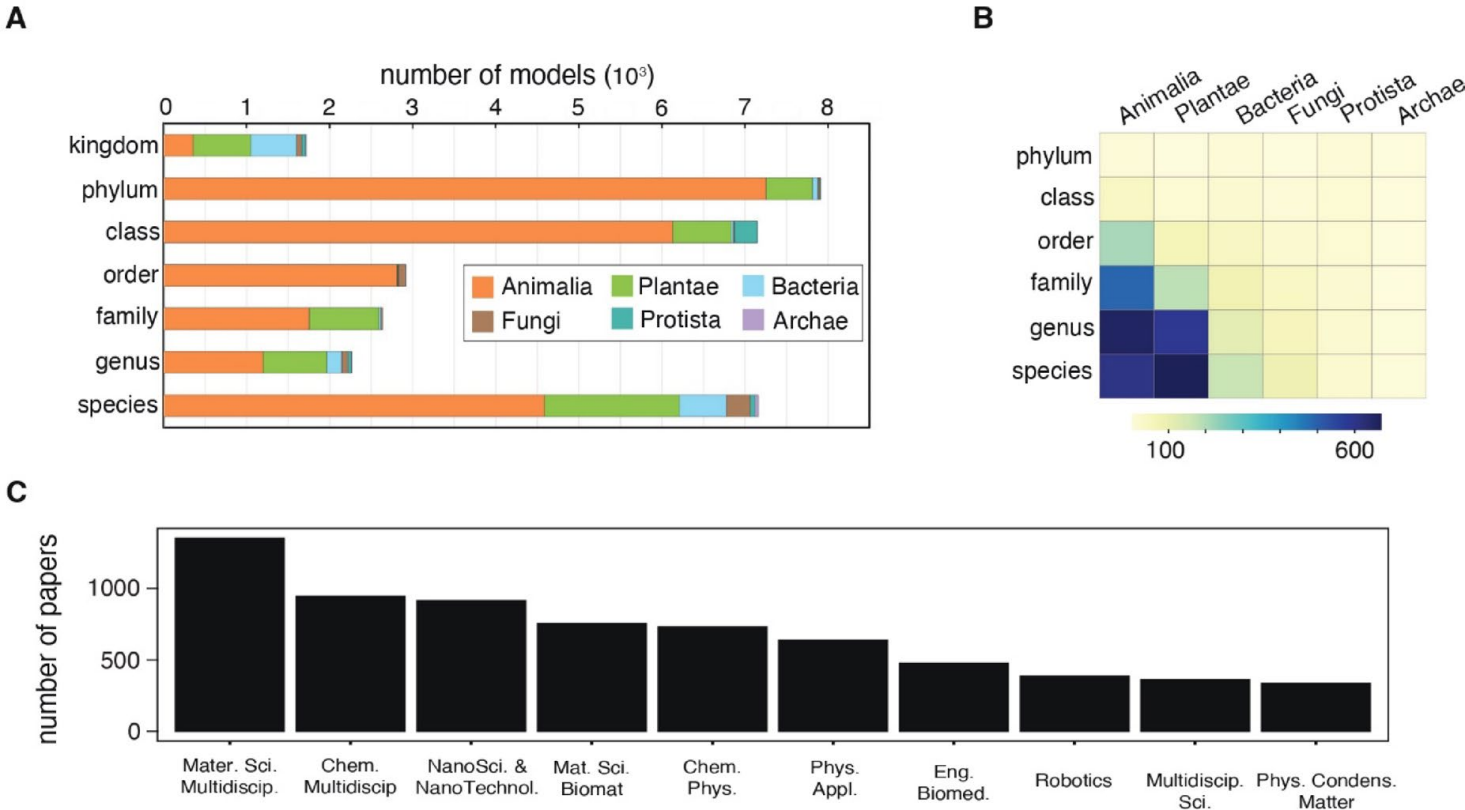
Taxonomic resolution and breadth of biological models, and their distribution across scientific disciplines. (**A**) Distribution of taxonomic resolution for identified models. Each model is classified solely at the taxonomic rank it was resolved (e.g., a family-level model is counted exclusively in the “family” rank and not in higher-level ranks). (**B**) Number of distinct taxonomic groups per kingdom across different taxonomic ranks. A species-level model contributes to its higher taxonomic ranks unless already counted (e.g., *Harmonia axyridis* and *Harmonia* both map to the same genus and thus add only one genus-level count). (**C**) The top 10 Web of Science categories for publications citing species-level models.

For all the biological models identified in this study, only 22.6% were specified at the species level (Fig. 3A). Intermediate taxonomic ranks were referenced less frequently (genus: 7.1%, family: 8.3%, order: 9.2%), whereas a substantial proportion of the cited models are at the class (22.5%) or phylum (24.9%) level. A smaller fraction (5.4%) was referenced at the kingdom level. To better capture the overall taxonomic breadth of biomimetic research, we calculated the number of distinct taxonomic groups rather than total model occurrences (Fig. 3B). Although kingdom Animalia had the highest total model count, kingdom Plantae exhibited greater species richness, with 679 plant species cited in biomimetic studies compared to 615 animal species. Moreover, Animalia, with 664 utilized genera, is the only kingdom in which a higher taxonomic rank shows greater diversity than a lower rank. Archaea remained the least represented group across all taxonomic ranks. This analysis also revealed 1,604 distinct species among the 7,164 models identified at the species level.

Among the 6,801 papers that explicitly reference species-level models, 2,093 distinct publication sources were identified—including journals, conferences, books, and reports—indicating the field’s broad dissemination. The five most prolific sources—*ACS Applied Materials & Interfaces*, *Advanced Functional Materials*, *Bioinspiration & Biomimetics*, *Scientific Reports*, and *Biomimetics*—account for 525 publications (7.7%), underscoring the highly dispersed distribution of research in the biomimetics field. An analysis of subject areas (based on WoS Categories) reveals that these papers span 174 disciplines. *Materials Science*,* Multidisciplinary* ranks first, followed by other leading interdisciplinary categories (Fig. 3C). *Biology* ranks 40th, and neither “biomimicry” nor “biomimetic” is yet recognized as a distinct WoS category (see Supplementary Material 2 for details). When ranking sources by the number of distinct species cited, the same five journals remain at the top, but in a different order: *ACS Applied Materials & Interfaces* takes the lead with 76 species, followed by *Bioinspiration & Biomimetics* (62 species), *Biomimetics* (57 species), *Scientific Reports* (55 species), and *Advanced Functional Materials* (44 species). A similar shift occurs in subject areas when analyzing the total number of distinct species cited rather than the publication count. *Engineering*,* Biomedical* and *Robotics*, initially among the highest-ranked fields, dropped out of the top ten, replaced by *Biochemistry & Molecular Biology* and *Engineering*,* Multidisciplinary*. This suggests that *Engineering*,* Biomedical* and *Robotics* are comparatively conservative in their choice of biological models.

Cross-referencing biological model data with corresponding publications in WoS enables more detailed analyses of model-application relationships. Here, we highlight three case examples: (i) humans, the most frequently cited species-level model; (ii) geckos and (iii) spiders—two classic models frequently used in biomimetics. (i) With 2,426 model counts, *Homo sapiens* is the most cited species in biomimetic research, appearing in 2,399 publications. The top five sources citing *Homo sapiens* include *Advanced Functional Materials*, *ACS Applied Materials & Interfaces*, *Biomaterials*, *Advanced Materials*, and *IEEE Robotics and Automation Letters*. Cluster analysis of publication titles and abstracts reveals a strong association with robotics, control systems, and biomedical applications (Supplementary Fig. 1 in Supplementary Material 6). (ii) A total of 332 papers reference geckos. Leading publication sources include *Langmuir*, *ACS Applied Materials & Interfaces*, *Journal of the Royal Society Interface*, *Journal of Bionic Engineering*, *Advanced Materials*, and *Bioinspiration & Biomimetics*. Studies frequently mention terms such as “attachment”, “detachment”, and “dry adhesive”, underscoring a focus on adhesion-based studies; additional clusters indicate gecko-inspired climbing robots (Supplementary Fig. 2 in Supplementary Material 6). (iii) Spiders serve as biological models in 521 papers, with *ACS Applied Materials & Interfaces*, *Advanced Materials*, *Advanced Functional Materials*, *Chemical Engineering Journal*, and *Advanced Science* as the most common publication sources. Most studies center on terms such as “silk”, “mechanical property”, “toughness”, and “strength”, reflecting a focus on spider silk’s material properties (Supplementary Fig. 3 in Supplementary Material 6).

### Taxonomic bias in biomimetics

**Fig. 4:**
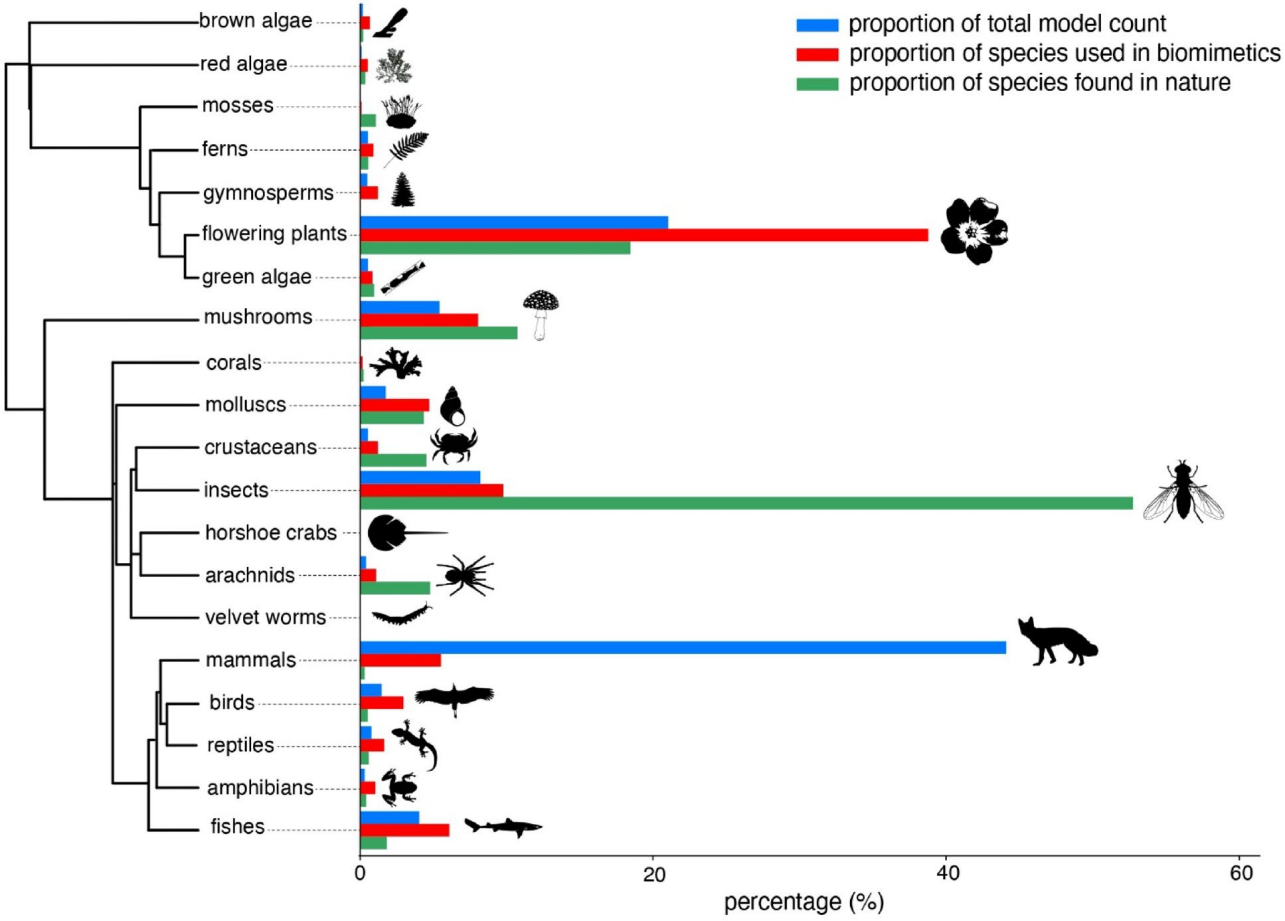
Phylogenetic distribution of biomimetic models relative to natural species richness. Bars represent, for each major taxonomic group, (i) its proportion of total biological model counts (blue), (ii) its proportion of species identified in this study (red), and (iii) its proportion of all described species (green). Taxa are arranged phylogenetically, illustrating disparities in biomimetic representation across evolutionary lineages. Phylogenetic relationships were obtained from timetree.org, and silhouettes were sourced from phylopic.org.

To assess taxonomic representation in biomimetics relative to described species diversity, we compared the species identified in this study to the total number of species described^31^ within each taxonomic group (Fig. 4). This exercise revealed considerable biases towards a few groups (notably mammals and other vertebrates), and against many others (e.g., insects, fungi, and arachnids). For example, insects constitute 52.8% of all known species but were represented by only 157 species—less than 0.015% of all described insects—in biomimetic research. Conversely, mammals, which account for just 1.3% of all known species, made up 44.1% of all species-level models in biomimetics (i.e., 89 mammal species contributing to 3,159 models). Birds, reptiles, amphibians, and fish—groups phylogenetically closer to mammals—were also utilized in biomimetics at a rate far exceeding their proportion among all described species (Fig. 4). Similarly, flowering plants represent 18.5% of all described species but account for 38.8% of species identified in biomimetics (622 out of 1,604 species).

### Are sources of inspiration for biomimetics drying up?

**Fig. 5:**
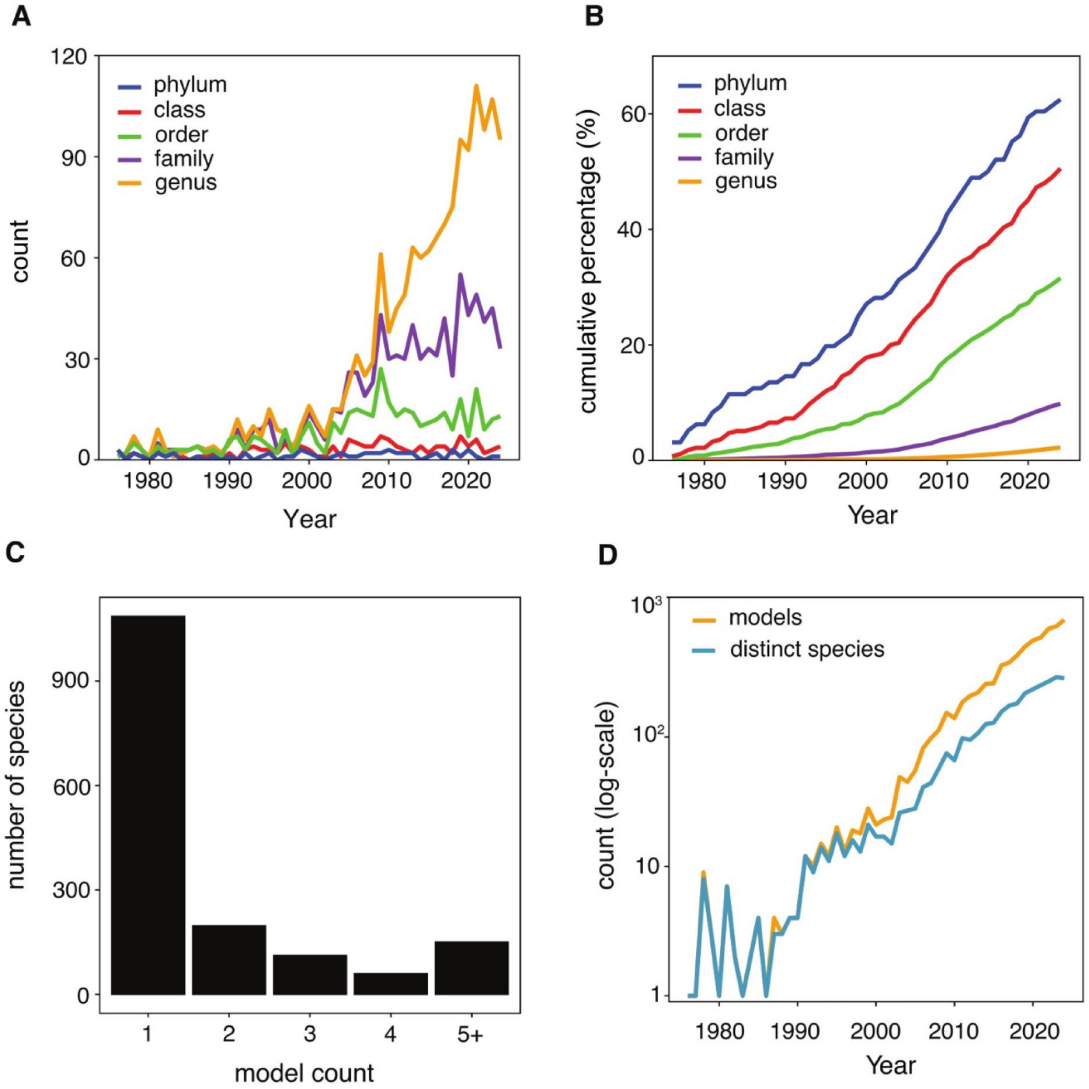
Research trends and distribution of biological models across taxonomic levels. (**A**) Annual incorporation of new taxonomic groups in biomimetics research at different taxonomic ranks (e.g., more than 110 new genera were introduced to biomimetics in 2021). (**B**) Cumulative taxonomic coverage over time, showing the proportion of all described taxa that have been used in biomimetics (e.g., over 60% of described phyla were cited by biomimetics up to 2021). (**C**) Distribution of species identified in this study based on their model counts, representing the number of species cited in different numbers of studies, from single-instance citations to species referenced in five or more studies. (**D**) Annual introduction of distinct species alongside their corresponding model count. Calculations were performed on a per-year basis, i.e. each year’s count includes all distinct species cited that year regardless of prior appearances.

To assess the pace and breadth of biomimetic research in exploring the treasuries of biodiversity, we examined how the number of distinct taxonomic groups has changed over time (Fig. 5A, B). The kingdoms were explored swiftly—by 1995, biomimetics had tapped all six of them at least once. Up to about 2008, new phyla, classes, orders, and families were steadily added to the biomimetic toolkit, but this increase leveled off afterward (Fig. 5A). The number of new genera added continued to rise until 2018 but has since shown signs of plateauing. Cumulative percentages (Fig. 5B) indicate that phylum and class have expanded the most rapidly, reaching 62.5% and 50.6% of their total observed diversity^32^ by 2024. Even a few newly referenced phyla or classes each year could push these values closer to full representation. In contrast, having drawn upon only 31.6% of all known orders, 9.8% of all families, and 2.2% of all genera, biomimetics has far from exhausted the wealth of inspiration offered by nature.

The majority (67.7%) of the 1,604 model species identified were referenced in only a single biomimetic publication. In contrast, a mere 150 species appeared in five or more publications (Fig. 5C). This underscores a strong reliance on a limited subset of species. Although the number of biological models used annually has increased substantially, the rate of new species incorporation has grown more slowly (Fig. 5D). This disparity has widened in recent years, with the ratio of total model count to distinct species surpassing 2.5 in 2024.

**Fig. 6:**
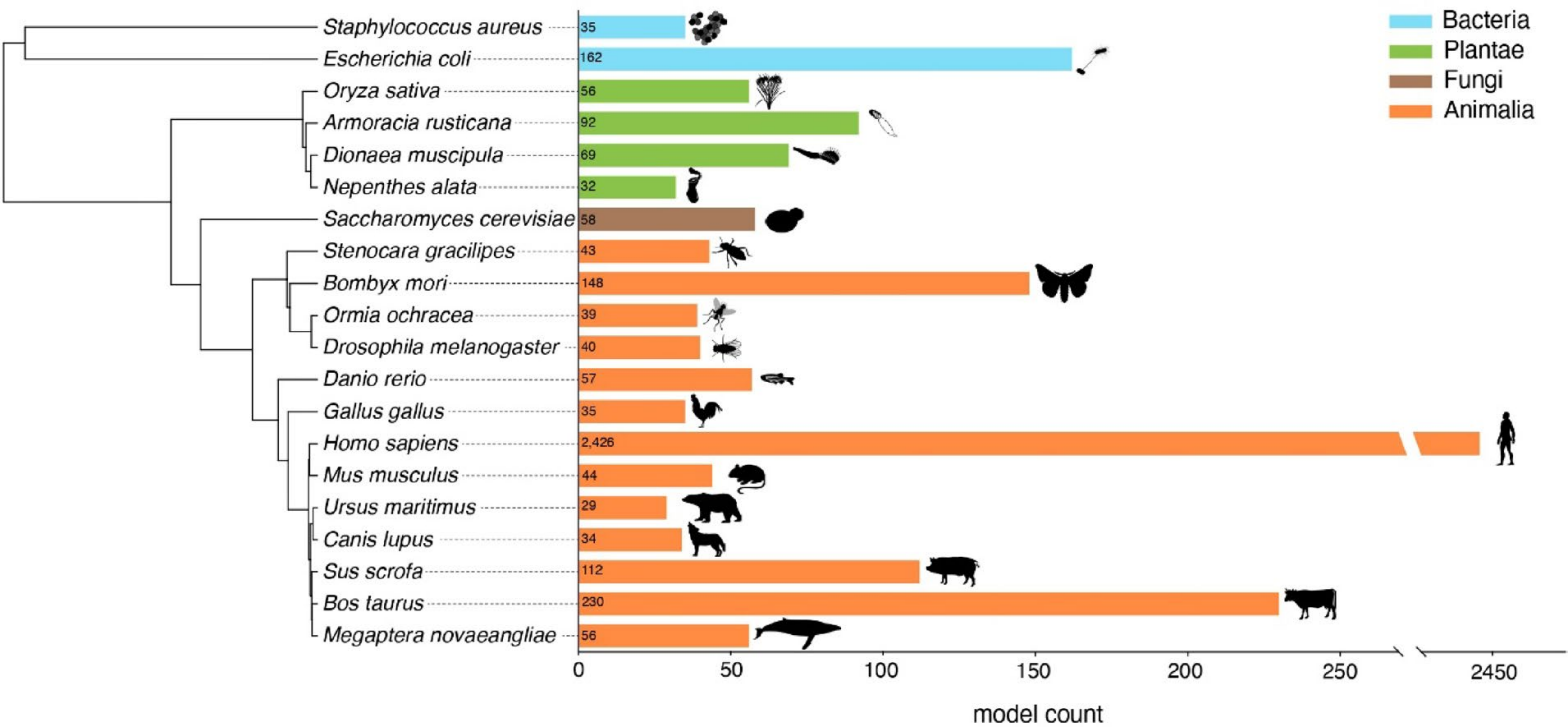
The 20 most frequently cited species in biomimetics and their phylogenetic distribution. This figure displays the phylogenetic relations of the top 20 species used in biomimetic research ranked by model count. Phylogenetic relationships were obtained from timetree.org, and silhouettes were sourced from phylopic.org.

Examining highly cited models, certain species have seen substantial increases in biomimetic use (based on the full list of identified models provided in Supplementary Material 3). For example, *Homo sapiens* had single-digit model counts before 2003 but exceeds 300 references in 2024. Similar trends are observed for cattle (*Bos taurus*) and silkworms (*Bombyx mori*). Among all species inspiring biomimetic research, humans are by far the most frequently cited, accounting for over one-third (2,426 models) of all species-level models—more than ten times that of the second-ranked species, cattle (*Bos taurus*). The top 20 most used species for biomimetics represent a diverse array of organisms, from fungi to humpback whales, with a strong dominance of animals (13 out of 20), particularly mammals (Fig. 6). However, plants and bacteria also feature prominently.

### Are comparative evolutionary approaches being adopted by biomimetics?

**Fig. 7:**
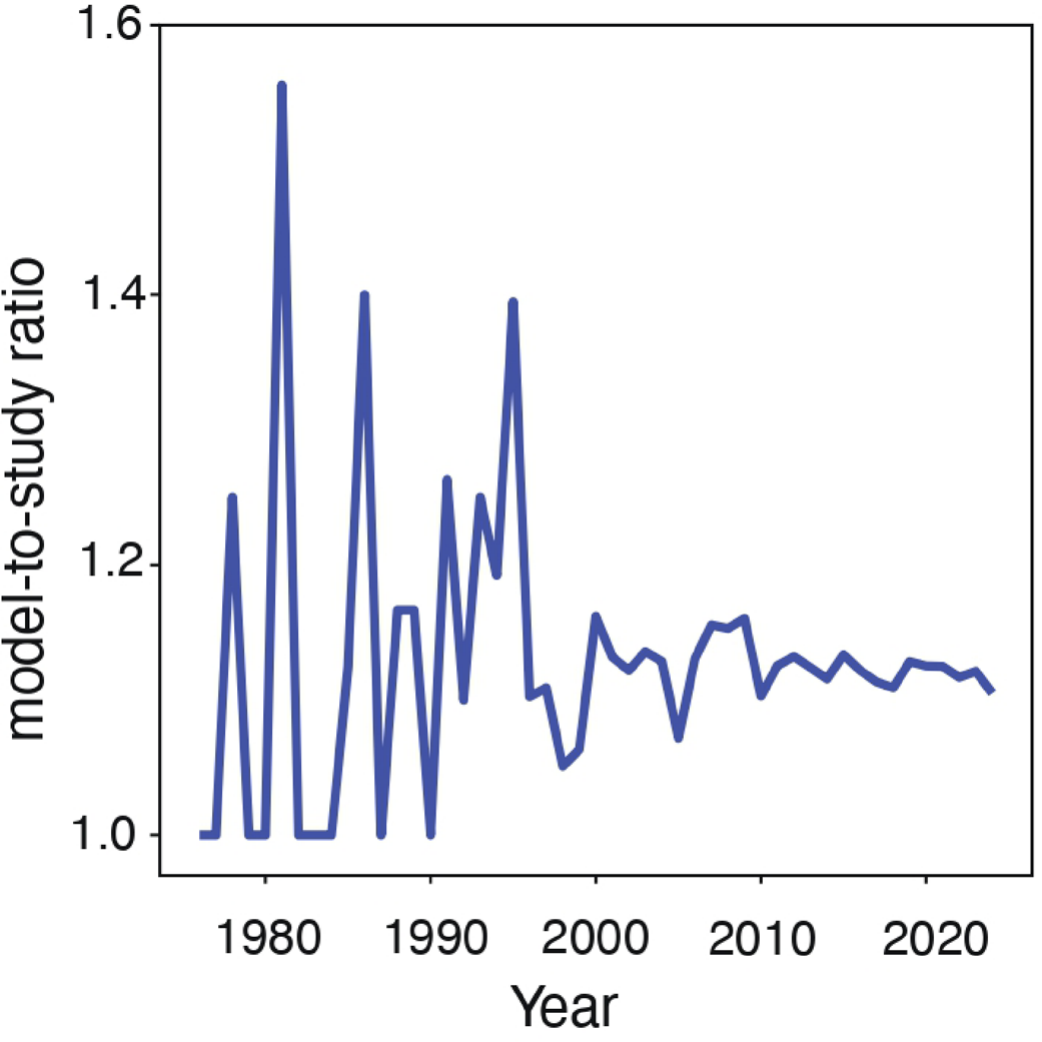
Annual trend in the average number of models cited per publication. Only publications with identified models were included in this calculation. Higher values indicate an increased tendency to reference multiple models within single studies.

Building on our examination of taxonomic diversity, we next investigated whether biomimetics leverages evolutionary perspectives—specifically, the comparative study of among-species variation and its relationship to environmental adaptation. The number of models cited per publication (those containing models) remained low, and this ratio stabilized around 1.1 since 2010, suggesting that the tendency to reference multiple biological models per study has not increased over time (Fig. 7). An analysis of all 28,333 publications that identified biological models revealed that fewer than 9% cited multiple models. To further explore the incorporation of evolutionary thinking in biomimetic research, we conducted a keyword search across these 28,333 publications. Surprisingly, fewer than 2% (556 publications) included at least one of the terms “comparative”, “convergence”, or “parallel evolution”. This result aligns with the low representation (0.06%) of *Evolutionary Biology* (WoS category) in these publications. Additionally, comparing the ratio of distinct species to total publications in each WoS category revealed further nuances in model diversity. For instance, *Materials Science*,* Multidisciplinary* cited 453 distinct species across 1,353 publications (a ratio of 0.33), and each of the top 10 fields in species count had a ratio below 0.5. In contrast, *Biology*, with 52 species in 56 papers, reached 0.93; while *Evolutionary Biology*, citing 17 species in 13 papers, exceeded 1.3. This comparison of species-to-study ratios suggests a stronger emphasis on applying diverse species in certain fields (see Supplementary Material 2 for details).

## Discussion

### Taxonomic patterns and implications for biomimetic research

Biomimetics has grown rapidly, driven by advances in foundational disciplines such as biology and engineering, as well as the increasing appeal of nature-inspired solutions across fields such as materials science and architecture^33–35^. However, our analysis reveals a critical disconnect: while the overall use of biological models and the proportion of publications citing them have steadily increased, the taxonomic diversity of these models has not kept pace. In fact, the rate of new taxon incorporation has plateaued or even declined in recent years. This discrepancy raises concerns that, despite the growing application of biological models, the field of biomimetics remains overly reliant on a narrow, familiar subset of taxa rather than exploring the vast, untapped biological diversity available in nature.

One key taxonomic pattern emerges at the kingdom-level distribution, where animals dominate biomimetic research. This imbalance likely stems not only from psychological preferences for mammals^21^ but also from the perceived adaptability and functional complexity of animals, which align well with various engineering applications^36,37^. Meanwhile, the steady growth of plant-based models reflects a more measured yet ongoing interest in botanical inspirations.

A select number of species have incited a disproportionate part of biomimetic research. Unsurprisingly, this includes humans and a small set of other mammals, but also a few lesser-known species, such as Venus flytraps (*Dionaea muscipula*), silkworms (*Bombyx mori*), and humpback whales (*Megaptera novaeangliae*). Elevating these species to model species can be beneficial, as any additional study will increase our knowledge of their biology and hence their value as biomimetic models. But it also bears the risk of fostering a type of taxonomic short-sightedness and missing out on opportunities offered by the myriad of untapped biological species and groups. For instance, while arthropods, fungi, and microbial systems have already proven to be valuable models for studies on material resilience and efficient energy use^38–41^, only a fraction of their species have come into the searchlight of biomimetics. Encouraging collaborations with biologists specializing in underrepresented taxa could help mitigate this imbalance. Our study quantifies how biomimetics currently draws on biological diversity, whether increasing model diversity—especially by incorporating unutilized organisms—will lead to improved project outcomes remains a promising avenue for future empirical research.

Another observed key pattern in biological model selection is the preference for higher-level taxonomic categories (e.g., phylum, class) over species-level identification. Our findings show that less than 23% of all identified models were resolved at the species level, highlighting a widespread reliance on broad taxonomic classification. While taxonomic rank does not necessarily reflect the scientific rigor of a biomimetic study, nor does a higher-level designation inherently diminish a model’s inspirational value, it does introduce ambiguity regarding the actual source of inspiration. Since biological models are central to biomimetics, precise identification is crucial. We acknowledge that biomimetic projects vary in complexity; in some cases, biological models serve as a conceptual spark rather than a direct blueprint for specific principles or metrics. In such contexts, citing a phylum or class (e.g., “bird” or “fish”) may suffice for a preliminary conceptual approach, particularly when abstracting general traits. However, relying solely on higher-level taxa risks oversimplifying context-dependent adaptations in later research phases. By contrast, species-level identification offers several advantages: (i) it allows researchers to link structural or functional traits to specific ecological conditions^42^; (ii) it enhances project rigor by encouraging a more precise understanding of the biological source, reducing misinterpretations (e.g., the Mercedes-Benz Bionic concept car was inspired by the boxfish, but later research showed that the species does not provide the expected low drag and adaptive stability benefits^43^); (iii) it improves reproducibility, ensuring greater clarity for future research.

Additionally, our analysis shows that over 90% of publications with identifiable biological models reference only a single organism. This pattern aligns with typical biomimetic workflows: solution-based approaches generally originate from a single organism’s principles, while problem-driven approaches first define a specific technical challenge before selecting the most suitable organism as the primary source of inspiration^44^. Although many successful biomimetic applications stem from an organism’s unique traits—and our data confirm that most research follows this single-model pattern—evolutionary biology demonstrates that convergent mechanisms can arise in diverse taxa^45^. For instance, geckos, spiders, and other arthropods all exhibit adhesive capabilities. Comparative analyses across multiple species can not only uncover underlying conserved functionalities but also provide alternative design pathways^46^. Recent studies further emphasize the benefits of integrating evolutionary perspectives, particularly when examining convergent evolution and how organisms adapt to distinct ecological pressures. This approach allows researchers to distinguish true adaptive features from non-functional traits caused by neutral evolution or genetic linkage, while simultaneously expanding the taxonomic breadth of biomimetic projects^21,28,47–49^. While comparative methods may not suit every biomimetic project, incorporating evolutionary perspectives where feasible could provide a competitive edge and lead to more innovative solutions.

### AI-powered text analysis

This study demonstrates that using GPT—a commercial large language model—combined with iterative prompts and structured processing can efficiently handle large-scale text analysis in biomimetics research. Unlike word frequency-based or other semantic analysis methods, GPT can interpret context and differentiate whether a term (e.g., “fish”) represents genuine biological inspiration or merely appears as a reference. As a result, it overcomes challenges such as the labor-intensive nature of manual processing, the ambiguity of high-frequency word analyses, and the difficulty of constructing an exhaustive biological name list for keyword matching. While training a custom AI model could improve accuracy, it would require substantial resources and specialized training data. By leveraging an existing, well-maintained model, this study achieved sufficient precision for large-scale text processing with moderate computational effort, illustrating the broad potential for AI-based tools in biomimetic research.

This study also highlights both the opportunities and the limitations of AI-driven analyses in biomimetics. While GPT’s output data met our predefined accuracy threshold, relying solely on titles, keywords, and abstracts (as well as manually validating only partial outcomes) introduces residual biases, particularly in taxonomic assignments involving broad or ambiguous model names. We anticipate that more capable AI models—paired with rigorously optimized prompts—will allow more accurate outcomes. Moreover, in light of the rapid evolution of both biomimetics and AI fields, rerunning this type of study in the future could yield increasingly rich insights, eventually allowing simultaneous retrieval not only of the biological models used but also of the specific properties under study (e.g., optical, thermal, mechanical) and the biological data types exploited (e.g., morphology, genetics, ecology).

### Outlook

Our systematic analysis of contemporary biomimetics literature shows that, despite the field’s rapid growth, it relies on a narrow set of “charismatic” taxa, often cites only higher taxonomic ranks, and typically references a single model organism per study. These findings raise critical questions about the underlying drivers of this selection bias and the strategies needed to mitigate it. Previous research suggests multiple contributing drivers, including inherent human preferences and established paradigms^24^, and limitations in widely used biomimetic tools^50^. Moreover, since the biological models used—particularly at the species level—represent only a fraction of the vast repository of biological knowledge, it remains unclear whether the observed bias is influenced by trends in other fields (e.g., biodiversity research, evolutionary or ecological studies, science communication, wildlife documentaries). Investigating these cultural and educational drivers could provide actionable insights for broadening the scope of biomimetic research.

Commonly proposed remedy strategies include greater involvement of biologists and expanding biomimetic databases with dedicated computational tools^19–21,23,28,50,51^, yet it remains uncertain whether these efforts alone can fully overcome entrenched biases. Building on prior strategies and our findings, we propose three additional recommendations: (i) Prioritize underutilized^19–21,23^ but well-researched taxa (e.g., insects, fungi) through interdisciplinary collaborations with biologists and ecologists. (ii) Emphasize species-level inspiration^42^ over higher taxonomic ranks. While broad classifications (e.g., phylum, class, order) can offer insights, considerable innovation potential lies at the genus and species levels, where an estimated 98–99% of taxa remain unexplored. Focusing on these lower ranks can reveal precise biological traits and unique adaptations that may be obscured when only broad taxonomic categories are considered. (iii) For suitable projects, incorporate evolutionary thinking to clarify the functional basis of biological models, or expand the number of analyzed models to identify common adaptations through comparative methods^28,52^.

In summary, overcoming the biases identified in this study will allow biomimetics to more effectively harness nature’s full innovative potential. Achieving this requires expanding taxonomical diversity^19–21,23^, refining model specificity^42^, and integrating evolutionary principles^28,52^. By embracing a broader range of biological inspirations, the field can unlock solutions as multifaceted as the biological organisms that inspire them.

## Methods

Our AI-driven literature search and analysis comprised four major stages: data collection, processing, validation, and results analysis (Supplementary Fig. 4 in Supplementary Material 6). The methods for each stage are described in detail below.

### Data collection

In November 2024, we searched the Web of Science (WoS) Core Collection using the query “biomim* (Topic) OR bioinspir* (Topic)”. This yielded a total of 74,359 documents. To ensure comprehensive coverage of biomimetics-related literature, no additional filters were applied beyond the search terms. Titles, keywords, and abstracts of all documents were then extracted for GPT-analysis. The decision to analyze only titles, keywords, and abstracts was based on several factors. First, if the biological model is central to a study—often the case in biomimetics—it is expected to be mentioned in the title, keywords, or abstract. Second, given the large number of retrieved documents, resource constraints made full-text processing impractical. Third, limiting analysis to titles, keywords, and abstracts mitigates potential copyright or intellectual property concerns regarding uploading data to AI, especially with non-open-access papers. Finally, preliminary tests were conducted on several fully open-access publications by comparing full-text analysis with analysis based solely on titles, keywords, and abstracts. The results indicated that full-text analysis did not consistently improve accuracy and often introduced additional noise, i.e., the presence of numerous example species in the full-text as introductions instead of genuine inspiration sources led GPT to retain more irrelevant models. Naturally, our decision not to process full texts entails certain constraints: for example, an abstract may omit the exact species used as the biological model even though that information appears elsewhere in the article. We recognize this as a limitation of the present study and hope that, as AI models improve and AI-related regulations become clearer, future research will be able to overcome it.

To ensure data integrity, we performed a duplicate check of all 74,359 collected documents using DOI (Digital Object Identifier) matching (with a Python script) and identified 30 duplicates across 64 entries. Further manual verification confirmed that none of these documents contained any corresponding biological models, i.e., they will not affect subsequent analyses. The complete retrieved dataset from WoS is provided in Supplementary Material 1. Because our analysis draws only on limited input data (papers’ titles, keywords, and abstracts), we cannot, for such a large corpus, determine whether every paper strictly meets the definition of biomimetics as set out in DIN ISO 18458:2015^44^. We therefore chose to include all publications that feature biomimetics-related terms, aiming to analyze the most comprehensive dataset. As a consequence, the collection inevitably contains not only genuine biomimetics research but also works that could be seen as biomimicry, bioinspiration, and potentially even bionic or bio-utilization studies.

### Data processing

After data collection, we initiated analysis using GPT-4o-2024-08-06 API (OpenAI) via a Python script for data processing. The Python code with the specific GPT prompts is provided in Supplementary Material 4. Below, we outline the core logic of this approach and key considerations in the prompts’ design.

In addition to basic operations such as reading input texts, handling GPT output, and saving results in Excel format, the script performs two key tasks. First, it processes each of the 74,359 collected entries using a “model extraction” prompt to identify and extract any biological models cited as inspiration. Second, the script assigns taxonomic classifications (kingdom, phylum, class, order, family, genus, species) and common names to the extracted models using a “taxonomy assignment” prompt. A biological model is defined here as the organism that provides the biological inspiration for solving a technical problem within a biomimetic project.

Early iterations of prompt design revealed that splitting model extraction and taxonomy assignment into separate steps improved accuracy by reducing task complexity. Additionally, the data sent to GPT had to be separated into discrete items to ensure reliable processing. For example, when extracting models, sending multiple papers’ data in a single request resulted in lower accuracy and consistency compared to processing each paper’s title, keywords, and abstract individually with the full prompt.

The model extraction prompt identifies biological models directly relevant to a paper’s biomimetic research focus while excluding irrelevant mentions. It filters out cases where organisms are merely experimental subjects (e.g., lab mice in medical contexts) or cited as illustrative examples (e.g., burrs inspiring Velcro, lotus leaves for self-cleaning coatings, or geckos for adhesives—when cases are only cited to introduce the idea of bioinspiration in general but not as actual study models). Additionally, the prompt flags review articles for later filtering. To ensure consistency, it also consolidates redundant references within a study—for example, merging terms such as “spider”, “spider hair”, “gecko”, “geckos”, and “gecko feet” into distinct entries and only outputting “spider” and “gecko” as identified models.

The taxonomy assignment prompt classifies each extracted model into the standard biological taxon, from the kingdom to the species level. If multiple classifications are possible, GPT returns “NA”. For instance, “spider” can only be classified up to order level (Animalia–Arthropoda–Arachnida–Araneae) since it encompasses many families (e.g., Antrodiaetidae, Scytodidae, Salticidae), leaving the family to species levels as “NA”. To ensure consistency, the prompt instructs GPT to use the most widely accepted and up-to-date taxonomy, while avoiding more detailed subdivisions such as subspecies or subgenera.

### Data validation

To ensure data reliability, manual validation checks were performed for both the model extraction and taxonomy assignment steps to confirm that GPT-generated outputs in each step exceeded an accuracy of 95%. This threshold is the minimum requirement for proceeding with further analyses in line with this study’s objectives. Full details of the validation record are available in Supplementary Material 5.

### Validation of the model extraction outcomes

To validate GPT’s extraction of biological models, the first author of this study manually reviewed 100 randomly sampled entries of titles, keywords, and abstracts. The sampling followed a two-step randomization process: (i) the 74,359 entries (sorted by relevance in WoS) were divided into 74 blocks of 1,000, with the remainder forming a 75th block; one entry per block was randomly selected using an online generator (https://www.random.org/), yielding 75 entries; (ii) an additional 25 entries were randomly drawn from the entire dataset to reach 100 total samples. Each sampled entry was manually assessed for (i) whether the article is a review paper, (ii) the presence of a clearly utilized biological model (Y/N), and its identity. These manual evaluations served as the reference standard and were compared against GPT’s output in two key steps: (i) determining if GPT correctly identified review papers, and (ii) verifying the accurate extraction of biological models. Accuracy scores (ranging from 0 to 1) were assigned per entry based on the comparison outcomes (Supplementary Fig. 5 in Supplementary Material 6). Applying this validation workflow (Supplementary Fig. 5 in Supplementary Material 6) to the 100 samples yielded an average accuracy score of 0.959, supporting the reliability of the GPT’s results for further analysis.

### Validation of the taxonomy assignment outcomes

The second validation phase assessed GPT’s accuracy in assigning taxonomic classifications to extracted biological models. A validation sample of 100 entries was generated, consisting of the 33 biological models identified in the previous validation round and 67 additional entries randomly selected from the full dataset of 31,776 models using a random number generator (https://www.random.org/). Each sampled biological model’s taxonomic data was manually verified using databases including GBIF (https://www.gbif.org/), OneZoom Tree of Life Explorer (https://www.onezoom.org/), or Bioinspire-Explore (https://bioinspire-explore.mnhn.fr/explore). For each model, the corresponding taxa were obtained from the aforementioned databases by the first author and documented from the highest (kingdom) to the lowest (species) level, with “NA” assigned where multiple classifications were possible. The first listed name on Wikipedia was taken as the common name (e.g., *Bombyx mori* as “silkworm”). These manually assigned and documented taxa served as the reference standard for comparison with GPT’s outcomes. For each sample entry, an accuracy score was calculated (details of this calculation are provided in Supplementary Table 1 in Supplementary Material 6). The overall accuracy for GPT’s taxonomic assignment, averaged across all 100 sample entries, was 0.951, supporting the reliability of GPT’s output for further analysis.

### Data cleansing

Following the two validation steps, GPT-generated outcomes were deemed sufficiently accurate for further analysis. However, since the study’s subsequent analyses rely entirely on the taxonomy classifications, additional refinement steps were implemented to ensure consistency and accuracy. Two main procedures were applied:

1. Automated flagging and manual correction.

A Python script scanned all 31,776 extracted models for inconsistencies across taxonomic ranks (kingdom, phylum, class, order, family, genus, species). If GPT assigned the same lower taxonomic rank to different superior ranks (e.g., family Gobiidae classified under both Gobiiformes and Perciformes), the entry was flagged for review. All flagged entries were then manually verified against GBIF’s record (https://www.gbif.org/), and if an error was confirmed at a particular rank, a “snowball” method was applied: every model within the affected taxa (e.g., all entries classified under Gobiidae, Gobiiformes, and Perciformes) was systematically checked and corrected as needed. This approach ensured internal consistency across related taxonomic classifications.

2) Manual inspection of frequently used models.

We sorted the extracted models by their common names’ occurrence frequency in the dataset. All models with more than 40 occurrences were manually inspected and corrected if necessary, covering 182 common names linked to 18,435 models.

Through these two measures, all 31,776 biological models underwent automated validation, with approximately 60% receiving manual verification, further strengthening the reliability of the final taxonomy dataset used for analysis.

## Electronic supplementary material

Below is the link to the electronic supplementary material.

Supplementary Material 1

Supplementary Material 2

Supplementary Material 3

Supplementary Material 4

Supplementary Material 5

Supplementary Material 6

## Data Availability

All data needed to evaluate the conclusions in the paper are present in the paper and/or the Supplementary Materials.
