# Supplementary material for: Overlooked sources of inspiration in biomimetic research
Source: Sci Rep. 2025 Jul 15;15:25590. doi: 10.1038/s41598-025-11703-6 (PMC12263887; doi:10.1038/s41598-025-11703-6)
Supplement: Supplementary file 4 — Supplementary Material 4 [file 41598_2025_11703_MOESM4_ESM.docx]

#...

# Model extraction using GPT-4o API

def model_extraction_gpt1(text):

    response = client.chat.completions.create(

        model="gpt-4o",

        messages=[

            {

                "role": "system",

                "content": (

                    "You are a helpful assistant specialized in biomimetic research analysis. Your task is to process "

                    "published articles' information to determine the biological model(s) used in each paper. You identify "

                    "and classify biological inspirations mentioned in the input text and present them in a structured format."

                )

            },

            {

                "role": "user",

                "content": (

                    "Please follow these rules:\n"

                    "\n"

                    "1. **Extraction**:\n"

                    "   - Extract names of biological models (organisms), e.g., species or biological structures that are used as inspiration in the provided text.\n"

                    "   - Only include names that are central to the biomimetic study of the provided text.\n"

                    "\n"

                    "2. **Formatting**:\n"

                    "   - Provide the output **exactly** as a JSON array of dictionaries, with **no code fences, markdown formatting, or additional text**. The JSON should be the **only content** in your reply.\n"

                    "   - Example:\n"

                    "     [\n"

                    "       {\"Biological Model\": \"red blood cells\", \"NSB Flag\": \"NSB\", \"Review Flag\": \"review\"},\n"

                    "     ]\n"

                    "\n"

                    "3. **Specific Guidelines**:\n"

                    "   - **Single form**: Use single forms of biological model names when appropriate.\n"

                    "   - **Consolidation**: Consolidate similar biological models mentioned in different forms, such as spider, spiders, and spider legs only lead to one output: spider.\n"

                    "   - **Organism vs. Structure**: Extract only the name of the organism when specific structures of the same organism are mentioned. If specific biological structures are mentioned that are part of the same organism (e.g., \"starfish's ossicles, mutable collagenous tissues, and derma\"), only extract the name of the organism itself (e.g., \"starfish\").\n"

                    "   - **NSB Flag**: Set to 'NSB' if the inspiration is a structure, not a specific organism.\n"

                    "   - **Review Flag**: Set to 'review' if the article is a review.\n"

                    "   - **Exclusions**:\n"

                    "     - Do not include experimental organisms if not used for biomimetic inspiration. Such as mice used as experimental animal in a medical context.\n"

                    "     - Exclude any mentions of biological names used merely as examples or analogies and not as the true inspiration of the study itself. Such as the mention of gecko, lotus, or any other famous bioinpiration cases just as introduction.\n"

                    "\n"

                    "4. **No Valid Model**:\n"

                    "   - If no valid biological model is found, return:\n"

                    "     [{\"Biological Model\": \"NA\", \"NSB Flag\": \"\", \"Review Flag\": \"\"}]\n"

                    "\n"

                    "**Note**: Carefully think through the steps internally before providing the final answer.\n"

                    "\n"

                    f"**Text**: {text}"

                )

            }

        ],

        temperature=0.2,  # Lowered for more deterministic output

    )

    assistant_response = response.choices[0].message.content.strip()

#...

# Taxonomy assignment using GPT-4o API

def taxonomy_assignment_gpt2(text):

    response = client.chat.completions.create(

        model="gpt-4o",

        messages=[

            {

                "role": "system",

                "content": (

                    "You are an expert in biomimetics and biology, especially with taxonomy. Your task is to extract and understand the biological model "

                    "information from the provided input text. Identify and classify the biological inspirations mentioned into standard and well-recognized taxonomy"

                    "and present them in a structured JSON format."

                )

            },

            {

                "role": "user",

                "content": (

                    f"Please assign the following taxonomic information for the biological model mentioned in the text:\n"

                    f"**Text**: \"{text}\"\n"

                    "\n"

                    "Provide the output as a JSON array of dictionaries, where each dictionary contains:\n"

                    "- **Kingdom**: The highest classification into which living organisms are grouped (e.g., 'Animalia', 'Plantae', or 'NA').\n"

                    "- **Phylum**: Use the highest applicable level for the provided model, even if more specific terms exist. For example:\n"

                    "  - Use 'Chordata' for vertebrates, instead of subphylum, Infraphylum, etc.\n"

                    "- **Class**: The rank below Phylum, ensuring consistency across similar organisms. For example:\n"

                    "  - Use 'Mammalia' for all mammals, instead of subclass, Infraclass, etc.\n"

                    "- **Order**: The rank below class (e.g., 'Primates', 'Diptera', or 'NA').\n"

                    "- **Family**: The rank below order (e.g., 'Hominidae', 'Drosophilidae', or 'NA').\n"

                    "- **Genus**: The rank below family (e.g., 'Homo', 'Drosophila', or 'NA').\n"

                    "- **Species**: The lowest taxonomic rank (e.g., 'Homo sapiens', 'Drosophila melanogaster', or 'NA').\n"

                    "- **Common Name**: The common name of the species or organism, the most well-used regular names for the provided organisms (e.g., 'Human' for 'Homo sapiens', 'Fruit fly'for 'Drosophila melanogaster', or 'NA').\n"

                    "\n"

                    "If the biological model cannot be explicitly tied to a specific taxonomic level, set 'NA' for that level (e.g., spider can be assigned until order 'Animalia Arthropoda Arachnida Araneae', but can't be link to only one specific family, therefore family outputs 'NA').\n"

                    "\n"

                    "**Important**:\n"

                    "- Do not include any additional text or explanations.\n"

                    "- The JSON array should be the only content in your reply.\n"

                    "- Ensure that each level of taxonomy is distinct and without ambiguity or overlap between levels.\n"

                    "- Ensure taxonomy aligns with the principle of using the most standard and well-recognized taxon to maintain consistency across entries.\n"

                    "- Consistently apply the highest relevant classification level for each category.\n"

                    "- Avoid using more specific subclasses, subphyla, or suborder, etc.\n"

                    "- Provide 'NA' for any information that is not available or applicable.\n"

                    "- If the biological model refers to a structure or material (e.g., 'muscle', 'nacre') and not a specific organism, provide as much taxonomic information as possible based on the context.\n"

                    "\n"

                    "**Example**:\n"

                    "If the text is: 'spider'\n"

                    "Then the output should be:\n"

                    "[\n"

                    "  {\n"

                    "    \"Kingdom\": \"Animalia\",\n"

                    "    \"Phylum\": \"Arthropoda\",\n"

                    "    \"Class\": \"Arachnida\",\n"

                    "    \"Order\": \"Araneae\",\n"

                    "    \"Family\": \"NA\",\n"

                    "    \"Genus\": \"NA\",\n"

                    "    \"Species\": \"NA\",\n"

                    "    \"Common Name\": \"Spider\"\n"

                    "  }\n"

                    "]"

                )

            }

        ],

        temperature=0.2,

    )

    assistant_response = response.choices[0].message.content.strip()

#...
