# Supplementary material for: Overlooked sources of inspiration in biomimetic research
Source: Sci Rep. 2025 Jul 15;15:25590. doi: 10.1038/s41598-025-11703-6 (PMC12263887; doi:10.1038/s41598-025-11703-6)
Supplement: Supplementary file 6 — Supplementary Material 6 [file 41598_2025_11703_MOESM6_ESM.pdf]

Supplementary Materials for

“Overlooked sources of inspiration in biomimetic research”

Jindong Zhang*, Simon Baeckens, Raoul Van Damme, Kristina Wanieck

*Corresponding author: jindong.zhang@th-deg.de

This file includes:

Supplementary Text
Supplementary Figs. 1 to 6
Supplementary Table 1

Other Supplementary Materials for this manuscript include the following:
Supplementary Data 1 to 5

Supplementary Text

Analysis of the identified biological models' common names

By using the identified models' common names, we created a word cloud that reflects the most often-used models and their taxonomic rank distribution (Supplementary Fig. 6). Humans emerge as the most frequently referenced species in biomimetics, reflecting a strong reliance on human-based models. Other commonly referenced terms—including "spider", "fish", "mussel", "lotus", "gecko", and "butterfly"—reflect a tendency to focus on well-known taxa with distinct morphological or functional traits. Notably, most of these terms correspond to higher taxonomic levels (e.g., class or order), with few explicitly linked to a specific species. The relative scarcity of names with a large font-size in the word cloud further demonstrates that even among the organisms that have been used in biomimetics, some are repeatedly cited while others appear in only a few studies.

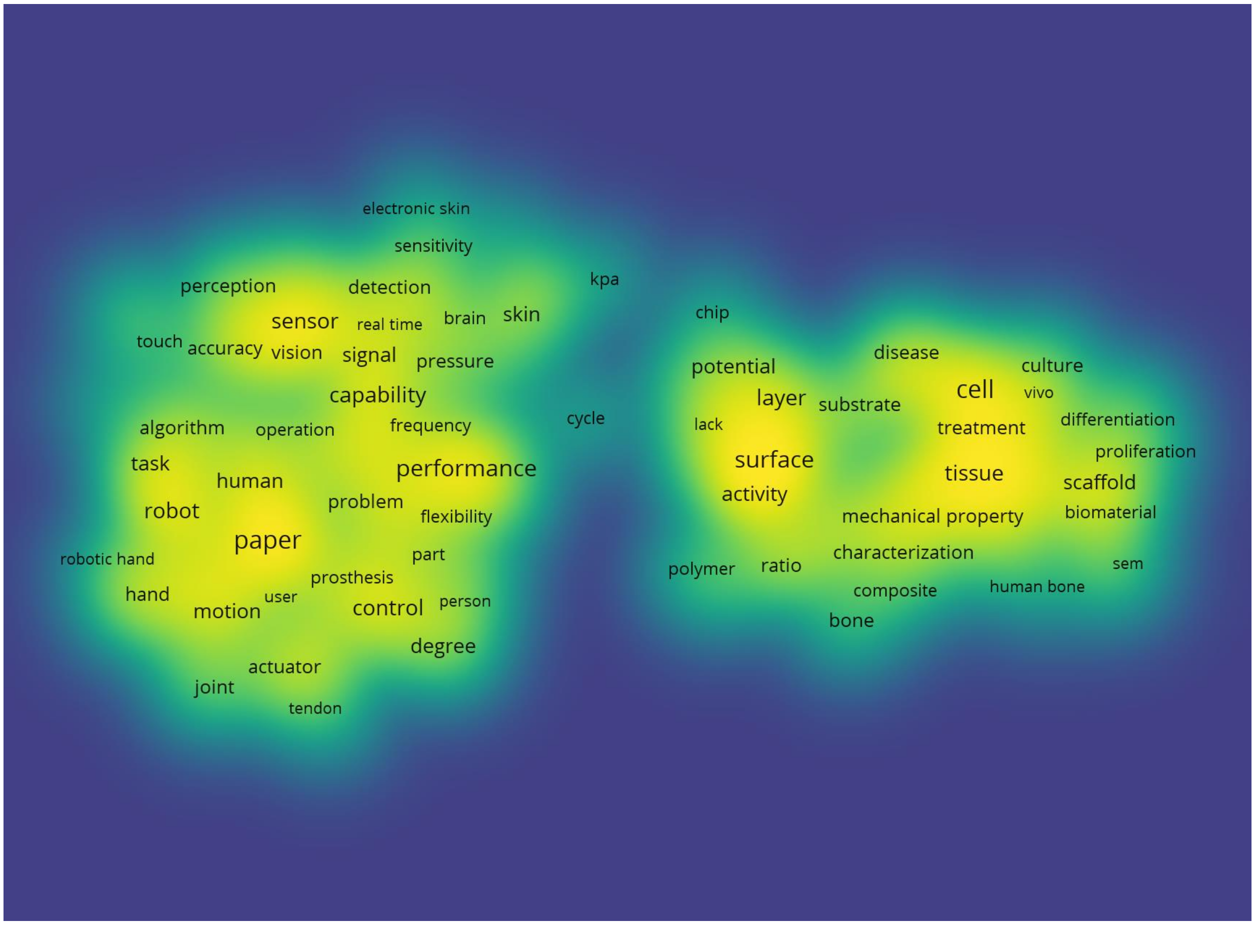

Supplementary Fig. 1. VOSviewer clustering of titles and keywords from papers using humans as the biomimetic model. Clusters highlight frequently co-occurring terms associated with human-inspired research.

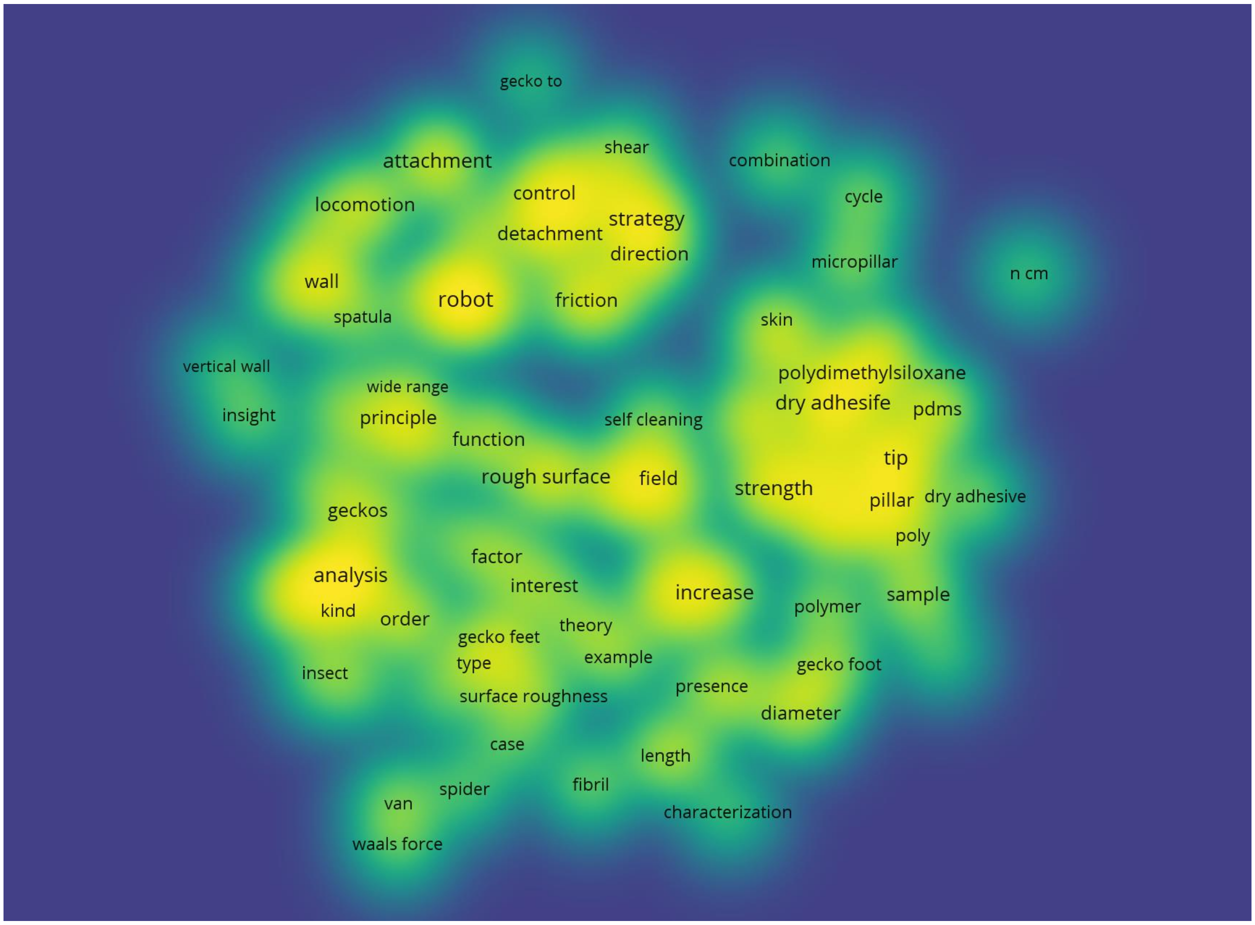

Supplementary Fig. 2. VOSviewer clustering of titles and keywords from papers using geckos as the biomimetic model. Clusters highlight frequently co-occurring terms associated with gecko-inspired research.

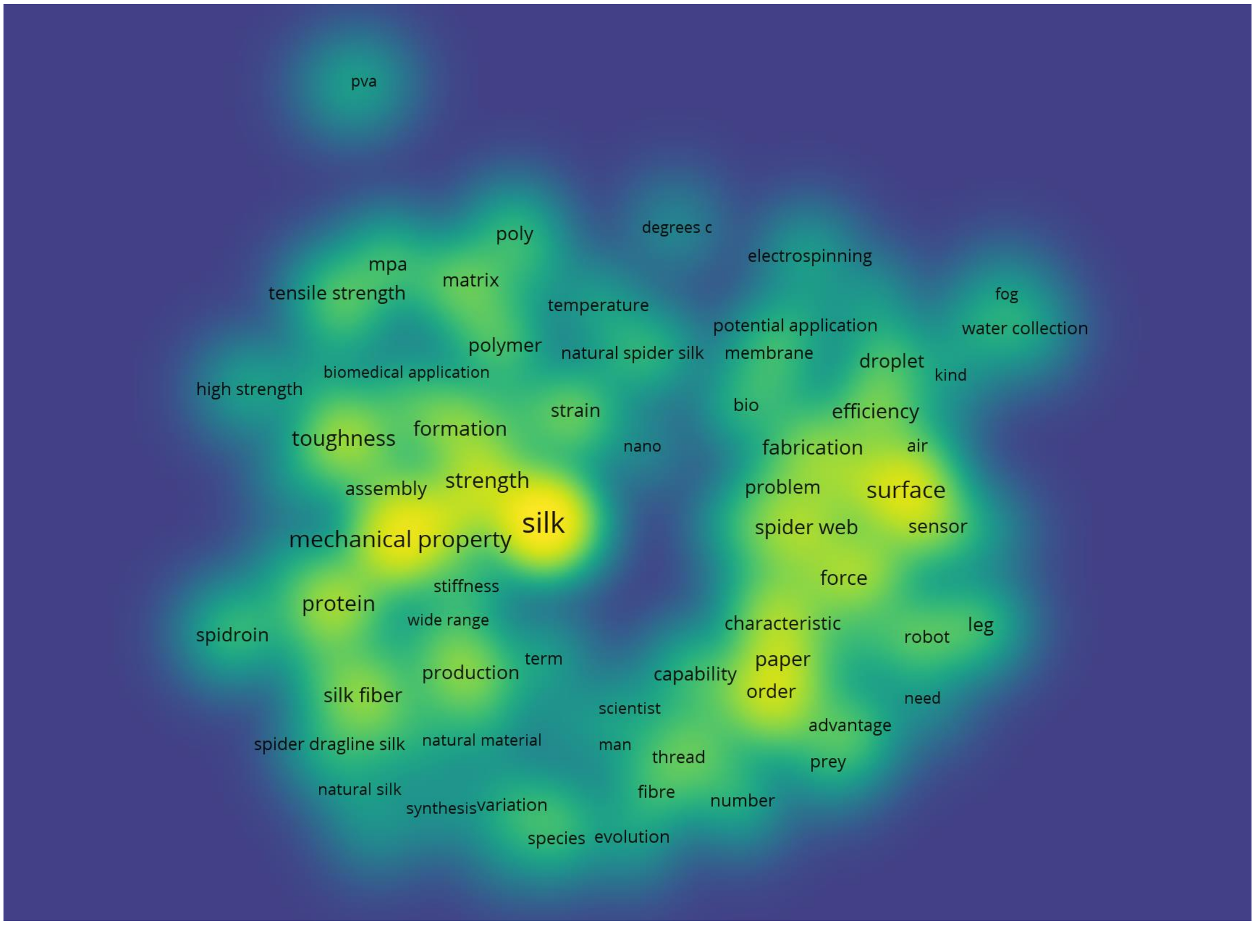

Supplementary Fig. 3. VOSviewer clustering of titles and keywords from papers using spiders as the biomimetic model. Clusters highlight frequently co-occurring terms associated with spider-inspired research.

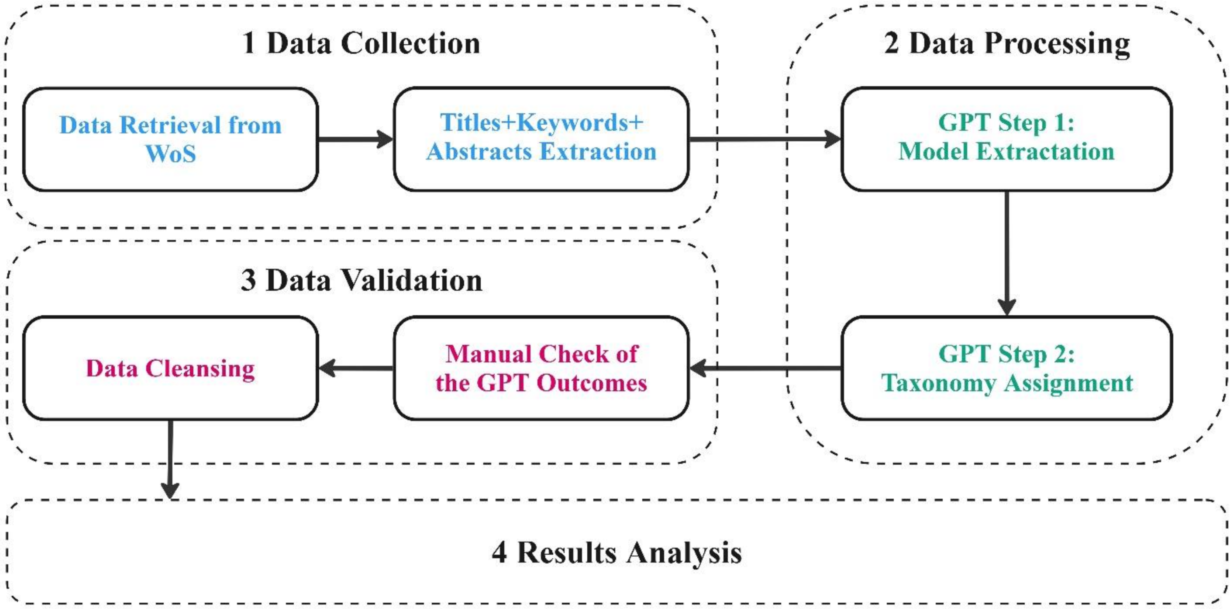

Supplementary Fig. 4. Overview of the methodological workflow for the AI-driven literature search. The process includes retrieving initial data from the Web of Science (stage 1), 2-step data processing using the GPT model (stage 2), and manual data validation (stage 3) for later result analysis (stage 4).

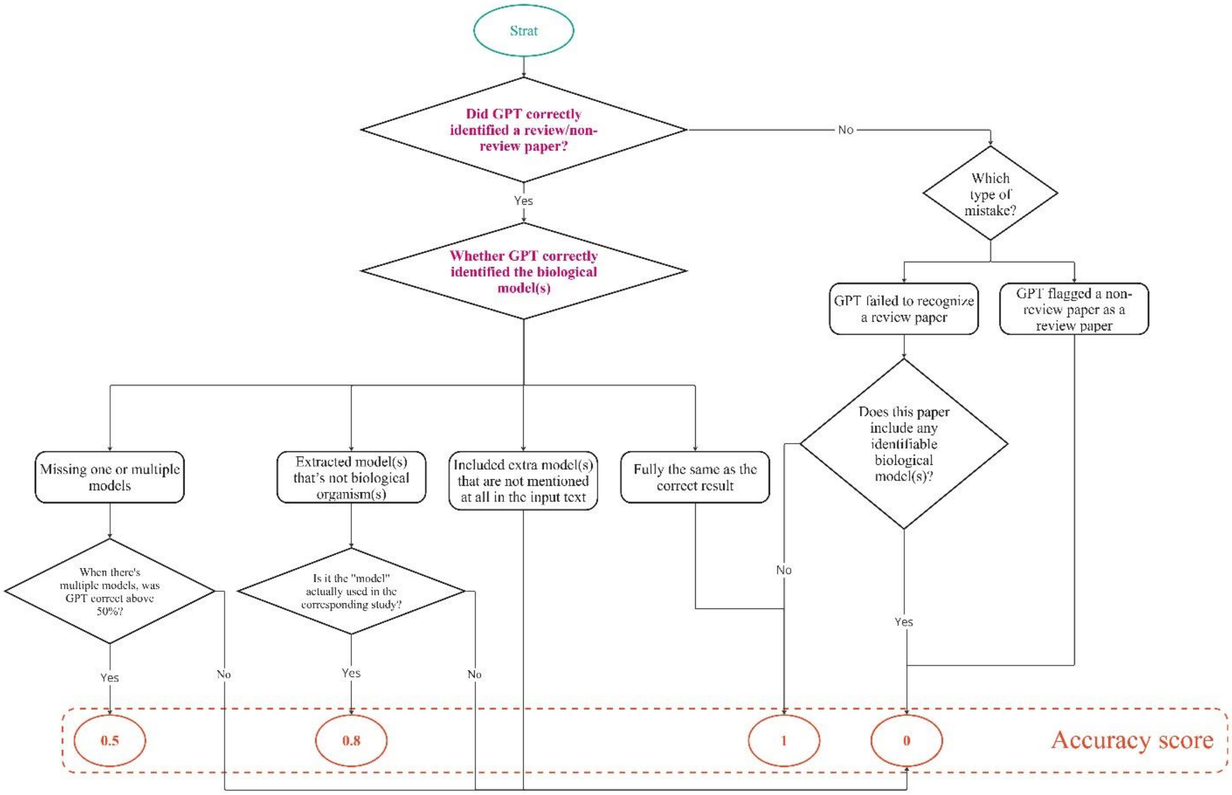

Supplementary Fig. 5. Procedures for evaluating GPT outputs and assigning the accuracy score. The accuracy score ranges from 0 to 1, where 0 indicates that GPT's output was entirely inaccurate and 1 indicates complete agreement with the manual assessment. The intermediate scores are assigned based on the value and reliability of the results for subsequent analyses. For example, a score of 0.5 is assigned if GPT correctly identifies a paper as a review/non-review, and accurately identifies more than half of the biological models (e.g., two out of three). Although not exhaustive, such partial accuracy can still inform further analysis. A score of 0.8 signifies correct recognition of the review paper and its declared models; however, those "models" refer to anatomical structures such as bones, hearts, or capillaries, rather than actual organisms' names. Although this information remains useful (heart can still be classified under class Mammalia in the later process), it does not merit the full score of 1.

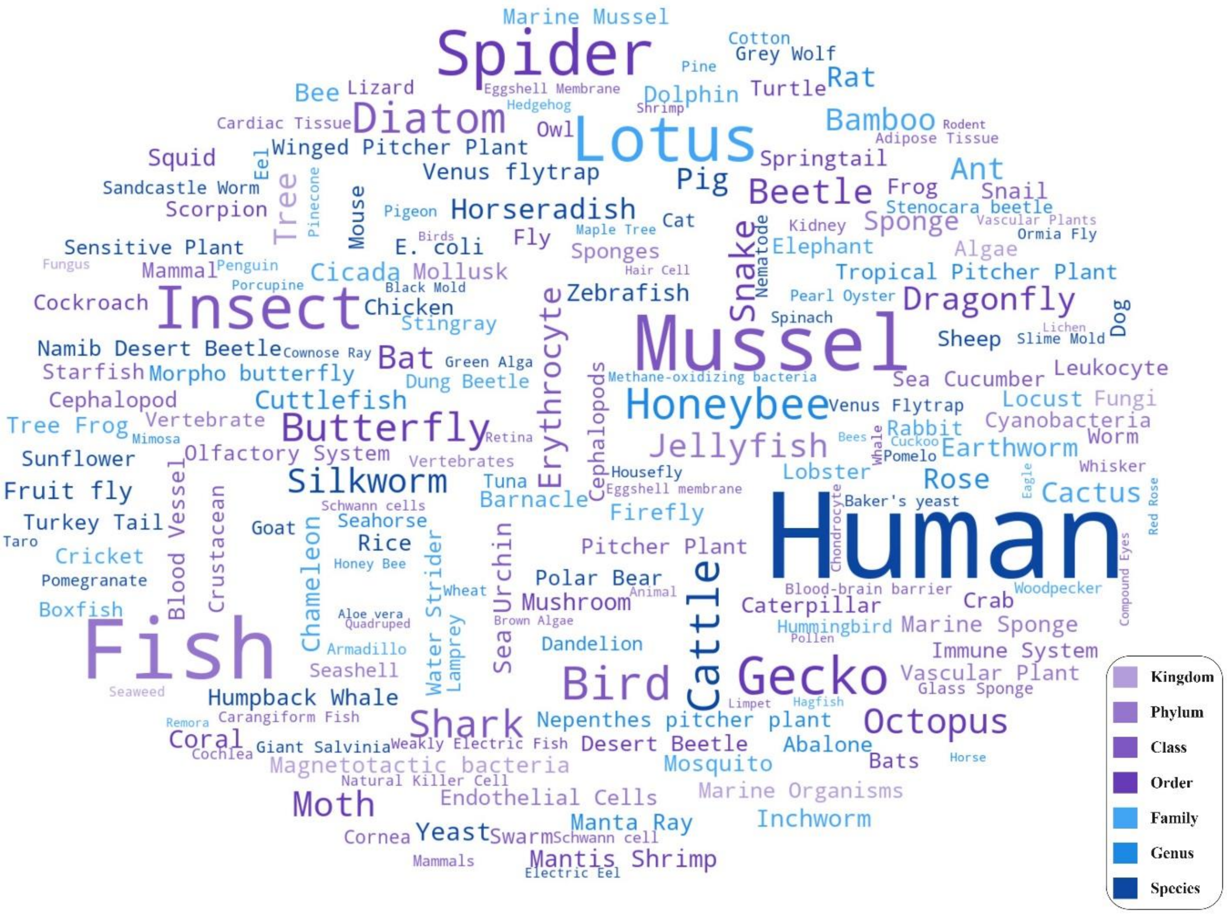

Supplementary Fig. 6. Word cloud of the identified biological models' common names. The color of the words corresponds to their respective taxonomic resolution. The size of the font reflects the model count: the larger the font, the more frequently that biological model has been employed in biomimetics.

	Kingdom	Phylum	Class	Order	Family	Genus	Species	Common Name
**GPT's results**	Protista	Amoebozoa	Myxogastria	Physarales	Physaraceae	*Physarum*	*Physarum polycephalum*	Slime Mold
**Correct results (Manual)**	Protista	Mycetozoa	Myxomycetes	Physarales	Physaraceae	*Physarum*	*Physarum polycephalum*	Slime Mold

Supplementary Table 1. An example of taxon assignment accuracy evaluation for *Physarum polycephalum*. For each sample entry, an accuracy score was calculated based on three criteria: 1) "taxonomic accuracy": the number of correctly identified taxonomic ranks divided by the total number of valid (i.e. non-NA) items. For example, in Table S1, GPT correctly classified 5 out of 7 valid ranks, resulting in a taxonomic accuracy score of 0.714; 2) "common name accuracy": assigned a score of 1 if GPT's common name matched the manual reference, otherwise 0. For example, GPT correctly identified "*Physarum polycephalum*" as "Slime Mold", yielding a score of 1; and 3) "overall accuracy": the total number of correct outputs (including taxonomic ranks and common name) divided by the total number of valid (non-NA) items; in Table S1, GPT's output was correct for 6 out of 8 items, yielding an overall accuracy of 0.75.

Supplementary Data 1. (separate file)

Initial retrieved data from Web of Science. The raw dataset of 74,359 records obtained via the specified search query ("biomim* (Topic) OR bioinspir* (Topic)").

Supplementary Data 2. (separate file)
GPT API Prompt. The custom prompt script detailing the instructions given to GPT for model extraction and taxonomic assignment.

Supplementary Data 3. (separate file)
Data validation record. The manual checks performed on a sampled subset (100+100) to verify GPT's outputs and the assigned accuracy scores.

Supplementary Data 4. (separate file)
Full record of identified models. A comprehensive table listing all extracted biological models, including their final taxonomic classifications and common names.

Supplementary Data 5. (separate file)
Full record of identified models' WoS categories and journal sources.
